# Fecal Short-Chain Fatty Acids as Potential Biomarkers for Attention-Deficit/Hyperactivity Disorder

**DOI:** 10.1192/j.eurpsy.2023.851

**Published:** 2023-07-19

**Authors:** N. Boonchooduang, O. Louthrenoo, N. Likhitweerawong, C. Thonusin, N. Chattipakorn, S. C. Chattipakorn

**Affiliations:** 1 Department of Pediatrics; 2 Cardiac Electrophysiology Research and Training Center; 3 Center of Excellence in Cardiac Electrophysiology Research; 4 Department of Oral Biology and Diagnostic Sciences, Chiang Mai University, Chiang Mai, Thailand

## Abstract

**Introduction:**

Growing evidence supports a possible link between gut microbiota and attention-deficit/hyperactivity disorder (ADHD) via the gut-brain axis. Short-chain fatty acids (SCFAs), the major metabolites produced by gut microbiota through anaerobic fermentation, may influence gut-brain communication.

**Objectives:**

To determine the alterations of gut microbiota and fecal SCFAs in children diagnosed with ADHD compared to healthy subjects.

**Methods:**

Fecal samples were collected from children with ADHD (n=10), and age- and sex-matched healthy controls (n=10) for gut microbiota and SCFAs analysis.

**Results:**

There were no significant differences in the abundance of any bacterial phyla in feces between groups. However, fecal concentrations of acetic acid, propionic acid, and butyric acid were significantly lower in children with ADHD compared to those of controls (**Figure1**). Interestingly, acetic acid and propionic acid levels were negatively correlated with ADHD symptoms (** Table 1**). Macronutrient and fiber intake, determined from food frequency questionnaires, did not differ between groups.

**Table 1.** The regression analyses predicting ADHD symptoms scores from fecal short-chain fatty acids level.

		*B*	*p*-value	95%CI	
Inattention score					
***Acetic acid***		-0.14	*0.009*	-0.24, -0.04	
***Propionic acid***		-0.18	*0.006*	-0.30, -0.06	
Hyperactive/Impulsive score					
***Acetic acid***		-0.10	*0.031*	-0.20, -0.01	
***Propionic acid***		-0.14	*0.018*	-0.25, -0.03	
Combined score					
***Acetic acid***		-0.12	*0.014*	-0.22, -0.03	
***Propionic acid***		-0.16	*0.008*	-0.27, -0.05

**Image:**

**Figure:**
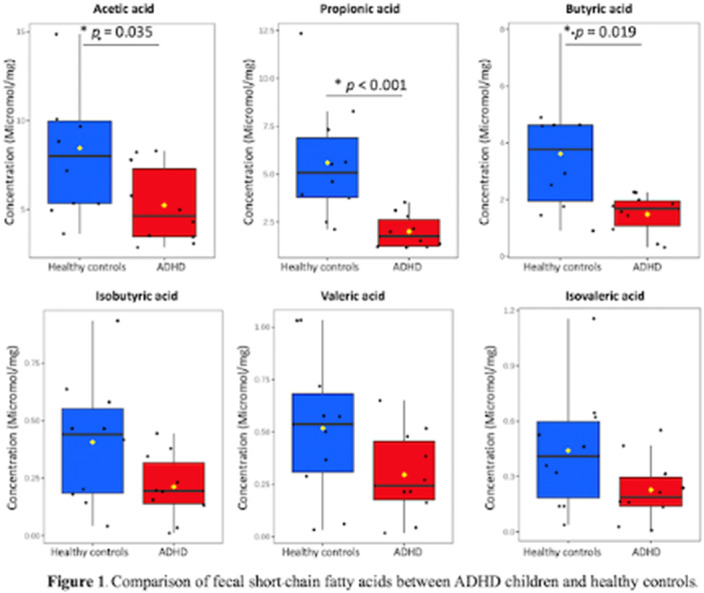

**Conclusions:**

Our findings suggested that gut dysbiosis was possibly developed in children with ADHD, as indicated by a significant decrease in fecal SCFAs. In fact, fecal acetic acid, propionic acid, and butyric acid may potentially be the early detector for ADHD. In addition, fecal acetic acid and propionic acid could be potential biomarkers for the severity of ADHD.

**Disclosure of Interest:**

None Declared

